# Indian medical students in public and private sector medical schools: are motivations and career aspirations different? – studies from Madhya Pradesh, India

**DOI:** 10.1186/1472-6920-13-127

**Published:** 2013-09-15

**Authors:** Vishal Diwan, Christie Minj, Neeraj Chhari, Ayesha De Costa

**Affiliations:** 1 R.D. Gardi Medical College, Agar Road, Ujjain, India; 2 Global Health (IHCAR), Dept of Public Health Sciences, Karolinska Institutet, Tomtebodavägen 18 A, Stockholm 171 77, Sweden

**Keywords:** Motivation, Aspirations, Medical students, Public- private, India

## Abstract

**Background:**

In recent years, there has been a massive growth in the private medical education sector in South Asia. India’s large private medical education sector reflects the market driven growth in private medical education. Admission criteria to public medical schools are based on qualifying examination scores, while admission into private institutions is often dependent on relative academic merit, but also very much on the ability of the student to afford the education. This paper from Madhya Pradesh province in India aims to study and compare between first year medical students in public and private sector medical schools (i) motives for choosing a medical education (ii) career aspirations on completion of a medical degree (iii) willingness to work in a rural area in the short and long terms.

**Methods:**

Cross sectional survey of 792 first year medical students in 5 public and 4 private medical schools in the province.

**Results:**

There were no significant differences in the background characteristics of students in public and private medical schools. Reasons for entering medical education included personal ambition (23%), parental desire (23%), prestigious/secure profession (25%) or a service motive (20%). Most students wished to pursue a specialization (91%) and work in urban areas (64%) of the country. A small proportion (7%) wished to work abroad. There were no differences in motives or career aspirations between students of public or private schools. 40% were willing to work in a rural area for 2 years after graduating; public school students were more willing to do so.

**Conclusion:**

There was little difference in background characteristics, motives for entering medicine or career aspirations between medical students in from public and private sector institutions.

## Background

The medical education system in India is currently one of the largest in the world [1]. The British and the Portuguese introduced western medical education into India; in 1835 the British established the Madras Medical School and in 1840 the Portuguese started the Goa Medical College. University affiliated medical education became the norm after the 1850s with the opening of the first three Indian Universities in Chennai, Calcutta and Mumbai [2]. At the time of independence in 1947, western medical education in the country was very much organized and funded by the public sector.

In recent decades however, there has been a massive growth in the number of private medical schools in South Asia. India, whose private medical education system is one of the most rapidly expanding such systems in the world, is a prototypical example of market-driven growth in the medical education sector. While the number of medical education institutions in the public sector in India grew by only 36% in the period from 1970 to 2005, the number of private schools multiplied by a staggering 1,120% [3]. Public and private medical schools now produce equal numbers of medical graduates in the country. As per the Medical Council of India, there are now 194 private medical colleges as against 161 government medical colleges [4]. There have been a number of reasons postulated for the rapid growth of the private sector including public sector financial constraints [5], special interest groups in the private sector who saw an opportunity [6] and a market willing to pay for medical education [7].

Medical students in India enter medical school after 12 years of primary and secondary school education. Undergraduate medical education then leading to a degree of Medical Bachelor and Bachelor of Surgery is a 5.5 year course, including a year of rotating internship. Although under the law, private medical schools are required to be run only by not-for-profit bodies and charge a ‘reasonable’ tuition fee from students, the main source of income in these schools has been reported to be student fees [8,9]. While course admission into public medical schools is primarily based on academic merit, admission into private institutions is often dependent on relative academic merit, but also very much on the ability of the student to afford the education [10]. Thus there have been concerns that private medical schools make medical education accessible only to the more privileged [9,11].

Entry level examinations to medical courses are extremely competitive, both to enter public or private sector medical schools. Little is known about how medical students in private medical schools differ from those studying in public institutions in terms of motivations and career aspirations. It has been reported that in developing countries, students from private medical institutions are more likely to end up working in urban areas, specialized institutions or even emigrate [12,13]. Also, rural medical service is an often emphasized need in the country; though willingness among medical students to take this up varies. This study done in Madhya Pradesh province in India aims to study and compare between first year medical students in public and private sector medical schools (i) motives for choosing a medical education (ii) career aspirations on completion of a medical degree (iii) willingness to work in a rural area in the short and long terms.

## Methods

### Study setting

This study was conducted in Madhya Pradesh province, Central India. This is one of India’s largest provinces with a population of 72 million [14] spread over a geographic area of 304000 sq kms. The province has 6 medical colleges in the public sector and 5 in the private sector. Each year, the public sector admits 720 students and the private 600 students into the first year of medical education leading to the degree of Medical Bachelor and Bachelor of Surgery. This study was done among first year students in 9 medical schools (5 public and 4 private) of the province. Approval for the study was obtained from the ethics committee of the R.D. Gardi Medical College, Ujjain (no 120/2010).

### Data collection

The research team first met with the Deans/senior management of the colleges to explain the study and seek permission to interact with the students for this. Although all 11 medical schools consented to participate in the study, in two schools – one public and one private – the study was not conducted because of logistic reasons. First year medical students in each of the nine colleges were invited by a team of research assistants to fill an anonymous questionnaire. The research team met with students in their lecture halls, just after the end of a lecture. They introduced the study to the students, informed them that participation was voluntary and assured participants of anonymity. Written informed consent was taken from all participating students. Researchers were able to contact 792 students on the day of the survey in the nine colleges, none declined to participate. They were given a self-administered pre-tested questionnaire to fill. The questionnaire elicited information on background socio demographic characteristics of students, their motivations for being doctors, their willingness to work in rural areas and their long term professional aspirations. The study was done between September 2010 - January 2011, within a few months after students joined the course in Sept 2010.

### Data analysis

Descriptive statistics including means, medians and simple proportions have been used to describe background characteristics of the students and motives for entering medical school. Odds ratios are used to present associations between background variables and outcomes of interest, including willingness to serve 2 years in a rural area and willingness to establish a long term career in a rural area. For the former variable, a multivariate analysis was performed after significant associations were observed in the bivariate analysis.

## Results

### Participants

A total of 792 students in nine colleges provided responses. Background characteristics of students are presented in Table 1. Students were more likely to be from within the province and to have schooled in the English language. They were between 18–22 years of age, except for 8% who were older. The sex ratio of students was 3 men for every 2 women. A little over half of all students studied medicine at state owned (public sector) medical schools. A significantly higher proportion of students from outside the province studied in private sector schools compared to public sector ones (29% vs 8%, p<0.05). Also students in private medical schools were more likely to have a physician parent that those in public sector ones.

**Table 1:** Background characteristics of medical students in public and private medical schools in Madhya Pradesh, India

**Characteristics of students**	**Total (n=792)**	**Public (n=441)**	**Private (n=551)**	**OR (95% CI)**
Sex:				NS
Male	491 (59.5%)	268 (60.8%)	203 (57.8%)	
Age: (mean yrs, range)	20 (18–31)	20.5 (18–27)	19.8 (18–31)	
Schooled language:				NS
English	643 (81.2%)	353 (80.0%)	290 (82.6%)	
Hindi	149 (18.8%)	88 (20%)	61 (17.4%)	
Native of the province:	654 (82.6)	405 (91.8%)	249 (70.9%)	2.3 (1.8-3.1)
Physician parent	172 (21.7%)	79 (17.9%)	93 (26.5%)	1.3 (1.1-1.5)

**Figure 1:**
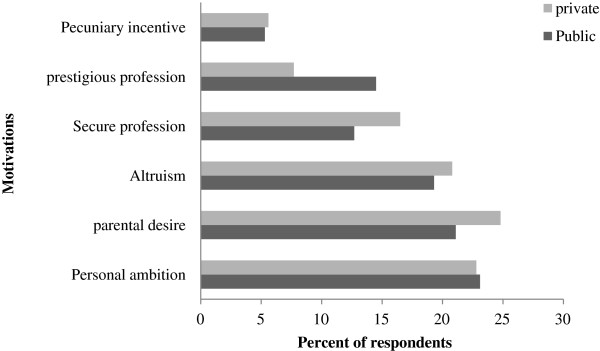
Reasons for studying medicine among respondents in public and private sector medical schools.

Approximately a quarter of all students chose medicine because of personal ambition. A similar proportion did because of parental desire. A further quarter entered because they regarded the profession as ‘secure’ or ‘prestigious’. A fifth of all students chose medicine for altruistic reasons (‘to serve’). Only a very small minority were motivated by a pecuniary incentive. Personal ambition, parental desire or any other motivation was not significantly associated with sector of school or any other background variable (Table 2). Reasons for choosing a career in medicine did not differ significantly between students in public and private medical schools. Figure 1 demonstrates the motivations of students opting for a medical career by public and private sector medical schools.

**Table 2:** Reasons for opting for a medical career

**Reason**	**N (%)**
Personal ambition	182 (23%)
Parental desire	180 (22.7%)
Service	158 (20%)
Security	114 (14.4%)
Prestigious profession	91 (11.5%)
Pecuniary interest	47 (5.9%)

The overwhelming majority of students (91%) wished to pursue a postgraduate specialist degree. Nearly 40% of students were willing to work in a rural area for two years after their graduation. Their motivations for doing so or not are indicated in Table 3. Over two thirds of students saw themselves establish careers in urban areas while a little over a fifth would practice in rural areas. Only a small minority of students saw themselves as having careers abroad.

**Table 3:** Aspirations of students

**Aspiration**	**Proportion**
Want to do a post graduate degree after graduating	
Yes	725 (91%)
No	67 (8.5%)
Willing to work in a rural area for two years immediately after graduation	
Yes	302 (38.1%)
For altruistic reasons	194 (64%)
To gain experience	55 (18%)
Earn money	44 (15%)
No	490 (61.9%)
Poor amenities	255 (52%)
No security	80 (16%)
No reason stated	144 (30%)
Preferred place of establishing career in the long run:	
Urban areas	509 (64.3%)
Rural areas	168 (21.2%)
Overseas	52 (6.6%)
Undecided	63 (8%)

Willingness to work in rural areas of the country in the short term and in the long term were analysed separately. The results are shown in Table 4. Public medical school students were more likely to be willing to work in rural areas in the short term (2 years) than private school students. However there were no other background variables associated with willingness to work in the rural areas for 2 years after graduation or to work on a long term rural career.

**Table 4:** Analysis of factors associated with willingness to work in rural areas (i) for 2 years after graduation (ii) as a long-term career

**Background characteristics**	**Association with willingness to do 2 year rural service**		Association with establishing a long term rural career*	
	***Unadjusted OR (95% CI)***	***Adjusted OR (95% CI)***	***Unadjusted OR (95% CI)***	***Adjusted OR (95% CI)***
Male Sex	1.18 (0.89-1.58)	1.16 (0.87-1.57)	1.41 (0-98-2.03)	1.39 (0.77-2.02)
Age^#^*≤* 22	0.84 (0.5-1.42)	0.78 (0.54-1.60)	0.88 (0.60-2.1)	0.93 (0.48-1.79)
Schooled in English	0.78 (0.54-1.12)	0.78 (0.54-1.13)	0.66 (0.44-1.02)	0.67 (0.44-1-03)
Native of province	0.85 (0.58-1.23)	0.75 (0.50-1.11)	1.22 (0.76-1.98)	1.27 (0.77-2.11)
Physician parent	1.22 (0.86- 1.72)	1.27 (0,89-1.8)	1.21 (0.80-1.84)	1.29 (0.84-1.97)
Public sector medical school	1.38 (1.04-1.85)	1.48 (1.09-2.01)	0.92 (0.65-1.30)	0.88 (0.61-1.27)

*comparison group= urban area.

^#^categorized into those ≤ 22 years and those >22 years.

A multivariate analysis controlling for the same variables as in Table 4 showed a significant association between public sector medical school and willingness to work in a rural area for 2 years remained significant (OR 1.48, 95% CI 1.09-2.01) when other background variables were controlled.

## Discussion

This is a first study from India reporting on medical students’ motivations and aspirations, in the context of the growing private medical education sector in the country. In recent years, India and other South Asian countries have seen a large expansion of private medical education [10]. Presently, 54% of all Indian medical schools are privately run [4], a trend that is likely to increase. There have been concerns expressed about the growing private sector presence in medical education in South Asia, that competition for students’ fees and an ineffectual accreditation process have resulted in questionable admission practices, stagnant curricula, antiquated learning methods, and dubious assessment practices [3].

Medicine is an extremely sought after professional education in India. More than a million students are expected to sit for the medical entrance test in 2013 [15]. All students answer a common test on which they are graded, these scores together with their pre-university exam scores weigh heavily to determine a students success. In public medical schools, these scores are often the only consideration; students who rank highest in these tests usually gain admission into public medical schools. Some students with high scores, who do not gain admission into public schools, may be admitted to private medical school as ‘government quota students’. The proportion of such students in a private medical school varies year on year by school. However a significant proportion of students in private schools are admitted based on a combination of test scores and their ability to pay high tuition fees and/or a high capitation fee.

While previous studies have indicated possible differences between students who enter public and private medical schools primarily in terms of paying capacity, there have been no studies thus far thus have clearly looked at differences in students that enter private and public sector medical schools in terms of background, motivations and career aspirations. A report from Nepal [12] studied career aspirations among medical students in Nepal’s private medical schools, but did not compare these with public school students. Overall though, our results were similar to the Nepal report in that a majority of the students wished to practice within the country, and in urban areas.

Results from our study from Madhya Pradesh showed no differences between the background characteristics of students in public and private medical schools, except that those in public medical schools were more likely to be from the province and those students admitted to private medical schools were more likely to have a physician parent. At the time of the study, private medical schools were also allowed to admit government quota students from out of province.

There was also no significant difference in the reasons which motivated these students to pursue a medical career. This is possibly because of the high level of competition that exists to enter medical school, selecting for similar students that enter both public and private schools. Also a proportion of students in private medical schools, could have been ‘government quota students’, who would therefore be more similar to public school students.

It was noted that a significantly higher proportion of students from outside the province studied in private sector schools compared to public sector ones. This raises the possibility that students from affluent backgrounds who have easier access to admission in private medical colleges are travelling to the province to pursue a medical career. The emergence of new private medical colleges in selected towns and cities has in given rise to an increasing trend of students travelling to and studying in private medical schools away from their home towns. It has been argued that the growth of private medical schools in South Asia has provided opportunities for students from the rising middle classes to be educated within the country, rather than pursue an off shore education [16].

Students from public sector medical colleges were more willing to work in rural areas after graduation. Factors such as having studied in a government secondary school, or hailing from a rural background have been shown to be associated with willingness to pursue a rural career in Nepal. Additionally; the study reported that students who received Ministry of Education scholarships requiring rural service were more likely to do so. In India, it has been often proposed that a short period of rural service should be made mandatory for graduates in public sector medical schools [17]. Reported barriers to rural practice include poor financial compensation, professional isolation, limited educational opportunities for self and family, and lack of specialty support. Less than half of medical students were willing to serve for a two-year period in a rural location, with public school students showing more willingness to do so. Factors such as having studied in a government secondary school, or hailing from a rural background have been shown to be associated with pursuing a rural career among medical students in Nepal [12]. The number of students who were willing to pursue a rural career in our study was too small to draw meaningful conclusions. A surprisingly small proportion in our study would consider a career abroad. An earlier study (2006) from South India [18] reported that 59% of students contacted wished to train or practice abroad. The numbers in our study are much lower, possibly given that our study was based in Central India. The government has recently introduced measures to curb permanent migration of Indian doctors overseas [19].

There are a number of limitations to our study. Our study is restricted to Madhya Pradesh, a large Central Indian province and hence the findings are generalizable to that province. Students in private sector schools in other provinces could have other characteristics. The views captured in our study are those of students who were present on the day of questionnaire administration. The views of those who were not present for any reason are not reflected. We surveyed first year medical students; it is possible that opinions and ideas could change towards the end of the five year course. We did not enquire into which specific ‘quotas’ students were admitted under eg. how many private sector school students were ‘government quota students’.

## Conclusion

In conclusion, despite there being many reports on the quality of infrastructure and human resources in private sector medical schools in India compared to public sector schools, there does not seem to be a significant difference in student characteristics, motivations or career aspirations between medical students studying private or public sector medical schools.

## Competing interests

The authors declare that they have no competing interests.

## Authors’ contributions

Conceived and design: VD, NC, AD. Performed the study: NC and VD. Analysis and interpretation of data: CM, AD, VD. Wrote the paper: VD, CM, NC and AD. All authors read and approved the final manuscript.

## Pre-publication history

The pre-publication history for this paper can be accessed here:

http://www.biomedcentral.com/1472-6920/13/127/prepub
